# Halloysite Nanotubes and Sepiolite for Health Applications

**DOI:** 10.3390/ijms24054801

**Published:** 2023-03-02

**Authors:** Giuseppa Biddeci, Gaetano Spinelli, Paolo Colomba, Francesco Di Blasi

**Affiliations:** Institute for Innovation and Biomedical Research (IRIB), CNR, 90146 Palermo, Italy

**Keywords:** halloysite, sepiolite, clay minerals, drug delivery, nanomaterials, biomedical application

## Abstract

The need for safe, therapeutically effective, and patient-compliant drug delivery systems continuously leads researchers to design novel tools and strategies. Clay minerals are widely used in drug products both as excipients and active agents but, in recent years, there has been a growing interest in research aimed at the development of new organic or inorganic nanocomposites. The attention of the scientific community has been drawn by nanoclays, thanks to their natural origin, worldwide abundance, availability, sustainability, and biocompatibility. In this review, we focused our attention on the studies inherent to the pharmaceutical and biomedical applications of halloysite and sepiolite, and their semi-synthetic or synthetic derivatives, as drug delivery systems. After having described the structure of both materials and their biocompatibility, we delineate the use of the nanoclays to enhance the stability, the controlled release, the bioavailability, and the adsorption properties of drugs. Several types of surface functionalization have been discussed, showing that these materials could be used for the development of an innovative therapeutic approach.

## 1. Introduction

Over the past few years as emerging materials, green and sustainable nanomaterials are increasingly become more and more popular. Among them, thanks to their numerous properties, naturally clay minerals with nontoxic, eco-friendly, and economic advantages have received increasing attention in both academic research and industries. Clay minerals, including one-dimensional (palygorskite, halloysite and sepiolite) and two-dimensional ones (kaolinite and montmorillonite), have special crystal structures and physicochemical properties. In fact, the nanometric dimensions of the material make it assume peculiar chemical–physical properties compared to conventional materials. They have been applied potentially in several fields as the substitution for toxic or expensive artificially synthesized materials [1,2]. Clay minerals and non-silicate minerals are commonly used as inactive ingredient, excipients, and active substances. These are used as excipients in pharmacological applications to improve the organoleptic, the chemical and physical properties. Due to their ability to swell in the presence of water and to buffer change of acidity, they are used as lubricants and agents to disperse easily and effectively active principles. Furthermore, their colloidal properties make them useful as gelling, emulsifying, and thickening agents. In addition to this, in recent years, they have attracted a lot of attention for the development of drug carrier and delivery systems by the hybridization of drugs with the clay minerals [3]. Some of the properties which make clay minerals useful in pharmaceutical applications are, for example, specific surface area, high adsorption capacity, chemical inertness, high cation exchange capacity, interlayer reactions, and low or null toxicity [3,4,5,6,7,8]. Due to the abundance in nature, low-cost and environmental friendliness, different target objects can be incorporated into one-dimensional clay minerals by surface modification or structural transformation to prepare functional materials. Furthermore, their biocompatibility, as detailed below, has been widely studied [9,10,11,12,13,14,15,16,17]. For these reasons, we decided to focus our attention on one-dimensional clay minerals such as halloysite nanotubes (HNTs) and sepiolite, that have been one of the research focuses of material and environmental sciences and biomedicine with a fairly considerable amount of data [18]. The aim of this review is to provide an overview on halloysite nanotubes and sepiolite structure, properties, and applicative aspects. Herein, we start with a brief background about one dimensional clay minerals, their biocompatibility and possible application in the biomedical field to provide new ideas to apply in research.

## 2. One Dimensional Clays Mineral: Sepiolite and Halloysite Nanotubes

### 2.1. Sepiolite

Sepiolite is a nanofibrous natural silicate with a micro fibrous morphology and typical particle size in the 2–10 µm length range that belongs to the clay mineral family [19] with light colour and low density and that has the chemical formula Mg_8_(OH_2_)_4_[Si_6_O_15_]_2_(OH)_4_·8H_2_O [20]. The dimensions of the sepiolite crystals, as summarized in Table 1, vary between 10 and 30 nm in width and 5 and 10 nm in thickness [21]. 

**Figure 1:**
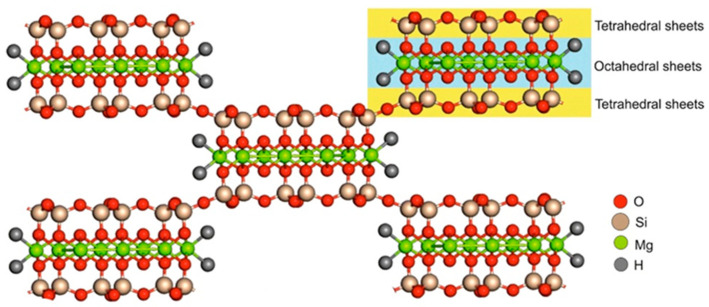
Sepiolite structure. Adapted from [30].

Usually, sepiolite occurs in two forms and is designated as α- and β-sepiolite. The first one is derived from hydrothermal alteration and is abundant in Tertiary rocks and Siliceous rocks, especially those rich in sulfates, carbonates, phosphates, salt, and zeolites. β-sepiolite is precipitated in various environments including pedogenic, lacustrine, lagoonal, and marine [22]. The main physicochemical properties of sepiolite, as, for example, the surface characteristics, show great variability depending on the geological origin of the silicate. The largest reserve of sepiolite is from deposits in Spain, followed by China, the United States, and Turkey [23]. In Spain, the most well-known sepiolite deposit is Vallecas-Vicálvaro on the outskirts of Madrid City. This deposit occupies approximately 7 km^2^ by forming sub-horizontal beds lenticular in shape and thickness ranging between 2 and 12 m [23]. In the deposit located in Mara, which was formed by sedimentary origin in the lacustrine basin, the sepiolite beds are composed of alternations of clays, marls, and carbonates, with the thicknesses between 10 cm and 1 m and averages of 50–60 cm [24]. In Turkey, marine-induced sepiolite deposits are located at the Mezgi ridge and sepiolite presents as 15–20 cm thick and is intercalated with siliceous dolomite units reaching up to 40–50 cm thickness [25]. Although the main physicochemical properties of sepiolite show great variability depending on the geological origin of the silicate, it is possible to affirm in general that sepiolite presents a needle-like structure and has talc-like layers that consist of two tetrahedral silica layers and a central octahedral magnesium layer as shown in Figure 1 [26]. The external surface is characterized by the presence of silanol groups (Si-OH), and the coordination of octahedral cations was completed by structural OH_2_ molecules [27,28,29]. The silanol groups and structural OH_2_ molecules represent the active hydrogen-bonding sides.

Sepiolite presents an alternating structure of blocks and tunnels that grow up in the fiber direction. The blocks are composed by a central magnesium oxide-hydroxide layer enclosed between two layers of tetrahedral silica [31]. Because of the discontinuity of the silica sheets, the silanol groups are present at the edges of the channels representing tunnels that are open to the external surface of the sepiolite particles [32]. Between channels and tunnels, it is possible to find coordinate and zeolitic water. The first one consists of water molecules that are bound to the Mg^2+^ ions located at the edges of the octahedral sheets. Zeolitic water, instead, is associated with the former ions through hydrogen bonding [31]. 

During the process of sepiolite formation, octahedral Mg(II) cations can be easily substituted by Al(III) and/or Fe(III); this may cause the generation of negative charges and formation of structural defects [33]. Furthermore, the substitution of tetrahedral Si(IV) cations by Al(III) may also occur [34] and that is why exchangeable cations exist in the channels to compensate for the structural negative charges. As results of its particular structure, sepiolite shows catalytic, rheological and sorptive properties, that make it ideal for several applications as, for example, a platform for the simultaneous delivery of different bioactive species such as lipids [35], proteins [36,37,38], polysaccharides [39,40,41] and virus particles [42]. Among the many properties, sepiolite possesses a stable intrinsic fluorescence. Therefore, by taking advantage of this natural fluorescence, it should be possible to select cells containing sepiolite just using conventional cell-sorting techniques. The International Agency of Research on Cancer (IARC) considered sepiolite as a non-hazardous and non-carcinogenic compound [43]. Thanks to its fibrous crystal morphology, sepiolite shows great potential in multiple applications, but the fibers of natural sepiolite usually present as aggregates or crystal bundles because of strong hydrogen bonding and van der Waals’ interactions among them. The aggregation consequently leads to the specific surface area and surface-active sites of sepiolite reduction, which limit greatly the applications of sepiolite as a fibrous nanomaterial. In order to use sepiolite as a functional material, it is very important to disaggregate sepiolite crystal bundles into individual nanofibers [1]. To summarize, is possible to say that sepiolite is a one-dimensional fibrous silicate clay mineral with a 2:1 layer-chain structure composed of two continuous tetrahedral sheets and one discontinuous octahedral sheet, that has several active surface groups and channels. The characteristic structure, together with the great specific surface area and active silanol groups makes it interesting for several applications. The sepiolite, similar to all clays, has the property of retaining and exchanging cations with the environment and this is known as cation exchange capacity (CEC) [44]. All these characteristics suggest that sepiolite could represent a very interesting starting building block for designing a wide class of nanomaterials for biological applications.

### 2.2. Halloysite Nanotubes

Halloysite is a two-layered aluminosilicate with a chemical composition similar to kaolinite with the chemical formula: Al_2_Si_2_O_5_ (OH)_4_·*n*H_2_O [45,46] and a hollow tubular structure in the sub-micrometre range [9,47,48]. In the hydrated form of halloysite, *n* = 2 in the formula. When one layer of water molecules is present among the multilayers, it is named “halloysite-(10 Å)”, where “10 Å” indicates the d_001_-value of the layers. When *n* = 0, the halloysite is named dehydrated or “halloysite-(7 Å)” [49,50]. Natural tubular halloysite clay has attracted a lot of attention in materials development because it is one of the few inexpensive nanomaterials available in thousands of tons at a low price [51]. Halloysite is defined as a 1:1 phyllosilicate and is characterized by a planar layer of tetrahedral silicates alternates with an octahedral geometry layer; these layers are bound together by oxygen bridges [52]. The halloysite nanotubes size may vary on the basis of the purification process they undergo and the extraction site, but they generally, as reported in Table 2, have an internal diameter of 10–30 nm, an external diameter of 40–70 nm [53], and a length from 200 to 2000 nm [54]. 

**Figure 2:**
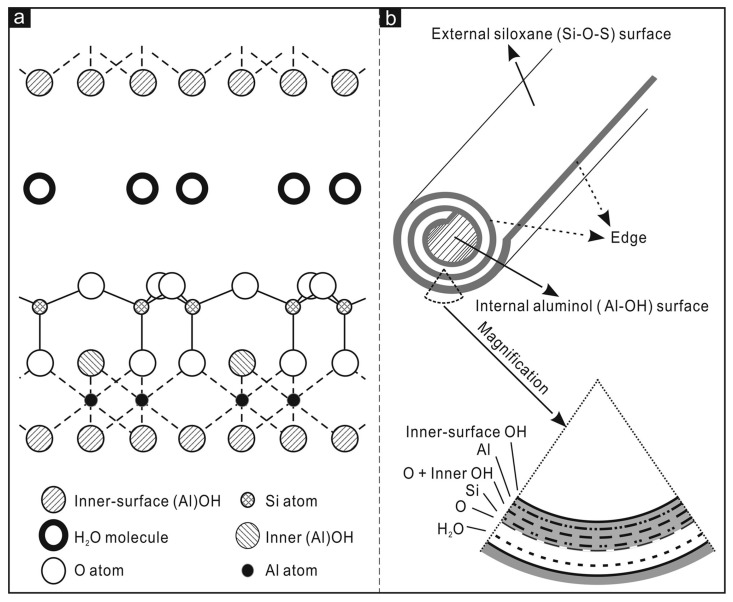
In (**a**) crystalline structure of halloysite-(10Å); (**b**) structure of a halloysite nanotube. Adapted from [61].

The siloxane groups are bonded via only one oxygen atom to octahedral rings at the outer part and the apical oxygen of tetrahedra becomes the vertices of octahedra [56]. One of the main characteristics of halloysite nanotubes is the different chemical composition between the outer and inner surfaces (Figure 2), in which there are, respectively, siloxanes groups (Si-O-Si) that give a negative charge and aluminolic groups (Al-OH) that give a positive charge [57,58]. These different charges are due to the different dielectric and ionization properties of silicon oxides and aluminium. When pH values are between 3 and 10, the positive charges are distributed in the inner lumen and the negative charges on the external surface. Principally, the tubule lumen is positively charged when pH ≤ 8.5, and the outer surface is negatively charged when pH ≥ 1.5 [59]. As a consequence of the tubular shape of halloysite nanotubes, on the outer surface are present only a few hydroxyl groups. For halloysite, it is possible to classify three types of Al-OH, according to their positioning on the surface, at the ends, and between the octahedral and tetrahedral sheets. All can be reactive and dissociate according to the pH of the solutions, except those placed between the octahedral and tetrahedral sheets, due to steric hindrance [60]. The inner lumen surface is covered by Al-OH groups and has a positive charge, and a functional group can be added by covalent modification. On the lumen surface it is possible to stably immobilize several organic groups. The external siloxane surface, with a negative charge, can be used to establish covalent bonding with molecules such as organosilanes [61]. Moreover, the outer surface can be modified by coating with cationic substances, such as polymers, biopolymers, and surfactants. This sort of modification can help to improve the dispersibility and biocompatibility of halloysite. Furthermore, the interstate surfaces can be modified by direct or indirect intercalation of small organic molecules and some monovalent cationic salts. This can lead to a weakening of the hydrogen bonds between the interstate and to increase in the surface among the various layers that may be considered as additional space for loading or adsorption [62].

Thanks to their numerous properties, their biocompatibility, and the possibility of functionalising the surface, halloysite nanotubes represent the ideal candidates for the development of new nanomaterials. 

## 3. One Dimensional Clays Biocompatibility

Thanks to their rheological properties, high interaction and high binding capacity with biopolymers, the role of mineral clays as a drug carrier has become the subject of extensive research in the last few years, showing a great potential for applications in biomedicine. To this end it is necessary to evaluate their toxicity in in vitro models as a first approach, to carry out a systematic analysis before their use in biomedical applications and to consider the possible consequential effects of their use on human health. In fact, the properties of nanomaterials, such as the size, surface area and zeta potential can modify their biological interactions compared to the raw materials [2,63]. An increasing exposure to different types of nanomaterials makes it essential to determine their possible negative impact on human health and potential toxic effects. Nanostructures, generally, once ingested, inhaled or topically administered, can be transported by blood and accumulated in various organs [64]. As a consequence of the entry into the systemic circulation, the absorption of nanoparticles by blood capillaries allows the distribution in several body districts. Depending on their surface characteristics, they can be recognized and degraded by macrophages [65] that are involved in the innate immune response and are specialized in the scavenging of foreign bodies in mammals and, for this reason, are widely used in toxicity assays [66,67]. As mentioned above, clays and clay minerals can be beneficial to human health working as active ingredients and excipients in pharmaceutical and cosmetic products [68]. It is necessary not to underestimate that clay minerals can have an adverse effect on human health, for example, when they are inhaled over a very long period. It has been reported that non-asbestos minerals belonging to fibrous silicates, such as sepiolite, raised health concerns about possible asbestos-like health effects. The toxicity of these minerals is generally related to both the geological conditions of formation or to the presence of quartz or asbestos from mining works [69]. 

### 3.1. Sepiolite Biocompatibility

Sepiolite, thanks to the fibrous structure, can transfer DNA into bacteria through the Yoshida effect, which was first described with asbestos. Friction forces by the fibers perforate the bacterial membrane, allowing DNA transfer via holes generated. The potential carcinogenicity of sepiolite results in the possibility to generate breaks into the bacterial genome inducing DNA damage and genetic instability [69,70,71]. However, as discussed by Castro-Smirnov et al., it is necessary to stress that there are strong differences between bacteria and mammalian cells, such as, for example, subcellular organization and size. Consider the fact that in eukaryotes, the genome is embedded into the nucleus, in contrast with bacteria [72], and that mammalian cells can also spontaneously expel sepiolite fibers [73]. Furthermore, it has been demonstrated that sepiolite is scarcely toxic at the doses used for mammalian cells transfection [73,74]. According to this, several studies have been carried out over the years demonstrating the absence of asbestos-like effects of the Tagus Basin’s sepiolite [75,76,77]. To describe further potential risks, Ragu et al. analysed how human cells (U2OS—osteosarcoma; GC92-SV40 transformed fibroblasts; GM03348-Primary skin fibroblasts) respond to interaction with sepiolite (from the Vallecas-Vicálvaro deposits), evaluating reactive oxygen species (ROS) production, inflammatory cytokines levels and genome integrity. The intracellular ROS levels were evaluated by flow-cytometry (FACS) and the results showed that sepiolite at a concentration of 5 μg/mL, induced the intracellular level of ROS, both after 24 and 48 h of contact and that the ROS level increased in a dose-dependent manner in the U2OS cells, suggesting that cells can detect and thus might respond to the presence of sepiolite. To investigate whether sepiolite triggers inflammatory cytokine production, the gene expression of some cytokines (IL-8, CCl2, IL1B, IL-6, IL-18 and TNFα) was measured by quantitative real-time PCR in U2OS cells exposed to two different concentration of sepiolite (10 and 50 μg/mL) for 24 and 48 h. After 24 h of exposure to sepiolite, only IL-8 gene expression was induced, while the expression of the other cytokines was induced after 48 h of exposure with both doses. So, it is possible to say that cells detect the presence of sepiolite and respond, leading the expression of inflammatory cytokines genes, according to different dose response. To assess the presence of DNA damage, cell cycle was monitored by FACS, showing that exposure to sepiolite did not trigger DNA damages response, cell cycle arrest and apoptosis. In summary, the results reveal that upon transient exposure, the cells can detect sepiolite and activate protective responses, not generating a substantial genotoxic stress. This could be related to the fact that the sepiolite fibers are located outside the nucleus [17]. The toxicity of unmodified sepiolite (in a concentration range of 1–10 μg/mL) was investigated on primary rat hepatocytes through the measurement of lactate dehydrogenase (LDH) activity in the extracellular medium after exposure time of 20 h. It was observed that there was no significant difference in the treated samples compared to the controls [75]. Sepiolite (from Tolsa Group, Spain) cytotoxicity was also investigated against HeLa (human cervical cancer) cells with a concentration of 450 µg/mL after 24 and 72 h of treatment. The results showed that after 24 h, cell survival is above 80% and that after 72 h there is a small decrease in the percentage survival, although it remained above 70%. Furthermore, the survival rate was measured in a dose-dependent manner, showing that the cell survival tends to decrease with the increase of sepiolite concentration, falling down to about 50% for concentrations around 1 mg/mL [78]. Natural raw sepiolite, as result of the agglomeration of its silicate microfibers, appears at the electronic microscope as bundles and this could influence the toxicity on mammalian cells. Brooks et al. in their work study the impact of sonication of a pure commercial sepiolite (Pangel S9) on haemolytic activity in blood cells and toxicity in U2OS (human osteosarcoma) and RG37 (human SV40-transformed fibroblasts) cells. The haemolytic activity of sepiolite, sonicated for 180 s, on erythrocytes drops to only 20%, unlike the non-sonicated that exhibits haemolytic activity values near 80%. The toxicity on U2OS and RG37 cells was evaluated after 24 h of exposure and results showed that sonication of sepiolite decrease the toxicity, indeed the frequency of living cells increases to levels close to that of untreated cells [15]. 

Taking into account that human cells are able to spontaneously expel sepiolite fibers [73] and that upon transient exposure, cells are able to detect it and to activate protective responses, it is possible to affirm that sepiolite does not exhibit significant toxicity and it is not a health risk. It is essential to note that the sepiolite fibers, according to the structural characteristics, can be used in different applications, the geological origin could affect other crucial parameters such as chemical composition [17] and the toxicity depends on the final equilibrium between internalization and externalization from the cells [15].

### 3.2. Halloysite Biocompatibility

The various interactions of halloysite nanotubes with living cells, encompassing electrostatic, van der Waals, and ion exchange, as well as cellular response, are critical in determining the behaviour of HNTs in the biological systems [79]. In a recent work, presented by Lazzara et al., the cytogenetic effects of four different halloysite clay nanotubes were evaluated, from various deposits in the world (HNT-N and HNT-U showed higher silica content; HNT-P and HNT-U had higher Fe_2_O_3_ concentration, and HNT-S had the highest amount of TiO_2_ and alumina content [80]). In particular, the cytotoxic and genotoxic effects were evaluated, respectively, through MTT (3-[4,5-dimethylthiazol-2-yl]-2,5 diphenyl tetrazolium bromide) and CBMN (cytokinesis-block micronucleus) assay on mammalian cell cultures (CHO, HeLa, HepG2). The results showed, in general, that at low doses and shorter times, HNTs showed low level of cytotoxicity. After incubation of 72 h it is possible to observe in HeLa cells an increase in cytotoxicity with HNT-U and HNT-S. HNT-U showed cytotoxic effects after 24 h at the highest concentration (600 µg/mL). In the HepG2 cells, HNT-U and HNT-S showed similar cytotoxic effects. These cells, when exposed to HNT-N, HNT-U, and HNT-P showed a decrease in cell vitality after 24 h, even if in the case of HNT-P cytotoxicity was not observed at 72 h of treatment [81].

The cytotoxic effects of HNTs were also investigated for short (24 or 72 h) and long-term (seven days) at doses ranging from 10 to 200 μg/mL on human alveolar carcinoma epithelial cells (A549) and human bronchial epithelial cells (BEAS-2B). The results showed that after 24 h of exposure, the IC_50_ value for HNTs in A549 and BEAS-2B cells was 152 ± 6.4 μg/mL and > 400 μg/mL, respectively. After 72 h of exposure, the IC_50_ values decreased to 49 ± 3 μg/mL in A549 and 45.1 ± 8 μg/ mL, in BEAS-2B cells. Thus, the results showed that cytotoxicity of HNTs depends on cell model, dose, and time of exposure [12]. It has also been observed that HUVECs (human umbilical vein endothelial) and MCF-7 (human breast cancer) cells show high cell viability after being treated with different concentrations of HNTs for 24 h. For both cell lines, vitality remains above 85% even when the concentration of HNTs reaches up to 200 μg/mL. When incubation times increase (48 h and 72 h), a slight decrease is observed in cell viability. In particular, at 72 h in HUVEC cells viability is approximately 60% at the maximum HNT concentration of 200 μg/mL [82]. Halloysite nanotube toxicity at several concentrations (10, 100, 500, and 1000 µg/mL) was further evaluated against human peripheral lymphocytes by means of mitotic index assay. The mitotic index assay revealed the inhibition of the lymphocyte’s proliferation only at the highest concentration (1000 µg/mL) [83]. The cytotoxicity of pure HNTs (0, 50, 100, 150, 200, 250 μg/mL) was also tested against CT26WT (murine colorectal cancer) cells and the results showed that after 24 h of treatment at 150 μg/mL, it is possible to observe a 25% decrease in cell’s viability and that this value continues to decrease with the increasing concentration of HNTs [84]. The in vitro effects of pristine HNTs were investigated on HeLa (human cervical cancer) and Raw 264.7 (murine macrophage) cells by MTS assay upon exposure to different concentrations (44, 88, 176 µg/mL). The viability was almost 80% for the highest concentration in HeLa cells and close to 100% in Raw 264.7 following a 24 h incubation. Even after prolonged treatments (48 and 72 h), cell viability remained high for both cell lines [13]. Taheri-Ledari et al. [85] performed evaluation of HNTs cytotoxicity, with a concentration of 50 μg/mL, on Caov-4 (human ovarian cancer) 3T3 (normal human fibroblasts) cell lines at different intervals of time (1, 6, 24 and 72 h). The viability was almost 100% for both cell lines even at 72 h [85]. Moreover, in the case of halloysite nanotubes, the cytotoxicity and biocompatibility studies demonstrate that these nanostructures are highly biocompatible, also in their modified version, indicating that they could represent ideal nanocarriers for the transport and delivery of drugs and biological molecules. However, to minimize the side effects, it is important to emphasize that certain factors, such as the choice of compound for functionalization, size and specific surface area must be taken into account [86]. 

## 4. Halloysite and Sepiolite for Biomedical Applications

The therapeutic effectiveness of a drug depends mainly on the concentration of the drug itself at the target site. In addition, it is very important to ensure optimal levels of the therapeutic agent at the target site and to maintain them for the duration of treatment [87].

Nano system drug administration involves the use of nanotechnology to deliver drugs to specific target sites within the body. This methodology is used to increase the efficacy of drug delivery by allowing the drug to be delivered in a more targeted and controlled way.

Nanoclays technology has the potential to revolutionize the healthcare industry and has attracted the attention of the scientific community, thanks to its natural origin, worldwide abundance, availability, biocompatibility, and sustainability [88,89]. These nano-sized particles have unique properties that could be used to create new treatments and therapies for a variety of illnesses and diseases. [90]. By using an adapted administration nano system, we can ensure accurate dosing of the therapeutic agent that can be released in a controlled manner over time. This ensures that the patient always receives the correct amount of the medication or treatment, reducing the risk of any adverse reactions [91]. Herein, we propose a general overview of these technologies using clays, which could be used to create delivery systems for drugs and gene therapy, targeted therapies for diseases such as cancer.

### 4.1. Drug Delivery

**Figure 3:**
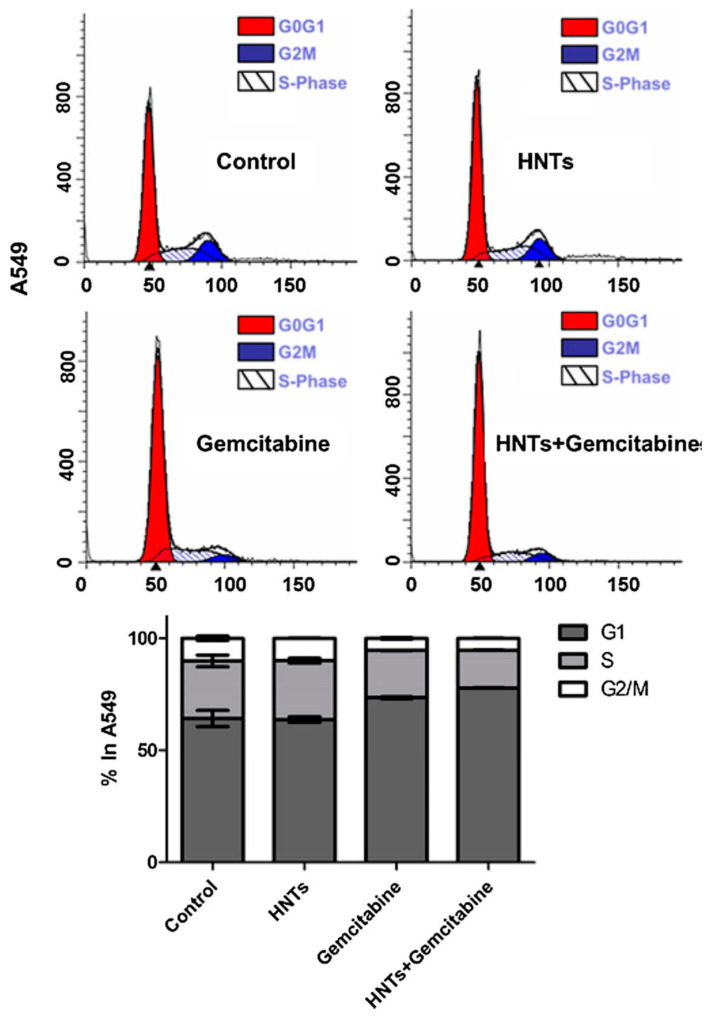
Study of A549 cell cycle after 24 h treatment with HNTs (0.1 µM), gemcitabine (0.1 µM) and HNTs + gemcitabine (0.1 µM). A549 cells were stained with propidium iodide. The red area shows G1 phase, the white area shows S phase, and the blue area shows G2/M phase. Bar diagram shows the % of cells present in different phases of cell cycle. Adapted from [105].

Owing to the special pore structure, large specific surface area and nontoxic side effects, sepiolite has been applied in the biological and biomedicine fields. Furthermore, due to its adsorption ability, sepiolite works perfectly as a sustained release agent for particular medicines, reducing the side effects of powerful drugs and helping to maintain the effective drug concentration in the organism for a long time [1]. Due to its structure, the internal surface of sepiolite is close to 300 m^2^/g, and the external surface ranges from 200 to 300 m^2^/g. This large interaction surface gives an understanding of how sepiolite can strongly interact with biomolecules [92]. Vitamin A (VitA) is an antioxidant, and it is an essential nutrient for the body and helps to protect cells from damage caused by free radicals. VitA plays a significant role in several physiological functions, but the poor solubility in water and the high chemical instability, lead to an easy loss of the physiological activity [93]. Thus, VitA encapsulation into sepiolite could be a promising method to improve its stability and bioavailability. In this regard, Calabrese et al. propose VitA loading by impregnation in sepiolite (from Tols) to study the in vitro release kinetics, aiming at delaying its photodegradation and allowing it to be absorbed more efficiently by the body. The results showed that the VitA release, under physiological pH mimicking conditions simulating the oral drug administration, was stable over time, suggesting the ability of sepiolite to prevent the oxidation process [94]. Chitosan/clay (CS/clay) composites, with clay particles acting as effective fillers, have attracted a lot of attention with the aim of achieving a synergistic effect, in improving physicochemical and drug release properties in the biomedical field [18,95,96]. With the aim of developing biohybrid materials for drug delivery, with enhanced adsorption properties, SEP (from Eskişehir, Turkey) has been combined with CS (CS/SEP) for the release of tetracycline (TC) in the treatment of patients for gastritis and peptic ulcers. In fact, TC is quickly destroyed by alkaline solutions and its potency is reduced in solutions with pH below 2 [97,98]. The results showed that the dispersed clay improved the thermal stability of the matrix, and that the increased content of clay in the composite causes a decrease in the release of TC due to the interaction of the silanol groups (-SiOH) of the clay and the hydroxyl groups (-OH) of the drug by the formation of the hydrogen bonding. The release study, carried out in aqueous medium, showed that the CS/SEP bio-nanocomposite films display a sustained release behaviour [99]. Praziquantel (PZQ) is an antiparasitic drug indicated for the treatment of schistosomiasis disease. Although it displays high efficacy and low toxicity, its low aqueous solubility requires high oral doses for its administration which gives side effects with consequent therapeutic noncompliance and the appearance of resistant forms of the parasite [100]. Borrego-Sánchez et al. have developed a release system to enhance the low solubility of PZQ by exploiting the ability of sepiolite (from Vicálvaro-Vallecas, Madrid) to encapsulate drugs. They present a methodology that uses organic solvents (ethanol, dichloromethane, acetonitrile) and clays, with the aim to prepare drug–clay complexes, to overcome problems of low aqueous solubility. The drug release profiles of the hybrid system compared with the pristine PZQ were evaluated in vitro in acid aqueous medium (pH 3) and in simulated intestinal fluid (pH 6.8) and the results showed that the dissolution rate of the drug was improved. In addition, they have also shown that the PZQ–SEPet revealed an increase in the solubility compared to the pristine drug and that the product was biocompatible with the HTC116 (human colon cancer) cells as it did not produce a decrease in cell viability or alterations in the cell cycle. According to the obtained results, these drug–clay complexes could represent a promising pharmaceutical system to improve the bioavailability of water low-soluble PZQ [101]. In several studies, HNTs have been used as nanocontainers or nanocarriers for drug delivery. Pristine halloysite nanotubes have been shown to establish weak interaction with the drugs and, for this reason, several methods of modification have been developed, for example, tubular entrapment, adsorption, or intercalation [102,103,104]. HNTs have been employed as vectors for the administration of various anticancer drugs. For example, gemcitabine (GEM) is the main drug used in the treatment of non-small-cell lung cancer. The mechanism of action of GEM consists in destroying the cells that are in the phase of DNA synthesis (phase S) but it can also block the cell cycle progression of cells from phase G1 to phase S. This drug is administered intravenously, but its action is non-specific and has inefficient biodistribution. The HNTs, thanks to the ability to cross the cell membrane [13] and to bind gemcitabine, can be used to overcome these limitations. In a study, it was demonstrated that HNTs loaded with GEM can block the cell cycle in A549 cells, with a reduction in the percentage of S-phase cells. This, for HNTs + gemcitabine, was a result of enhanced intracellular gemcitabine concentration, confirming that HNTs contribute to the intracellular transportation of the antitumor drug (Figure 3). Therefore, HNTs loaded with GEM are able to determine the inhibition of the cell division and growth of A549 cells [105]. 

**Figure 4:**
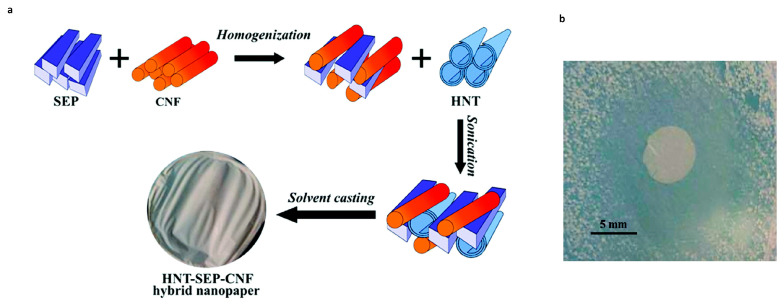
(**a**) Scheme for the synthesis of HNT–SEP–CNF hybrid nanopaper. (**b**) Inhibition zone of the salicylic-acid-loaded HNT–SEP–CNF hybrid nanopaper against gram-positive *S. aureus* at pH = 5.5. Images adapted from [113].

Doxorubicin (DOX) is one of the most used anticancer compounds among the anthracycline derivatives, because of the antitumor effect against most cancers such as breast, lung, brain, lymphoma and leukemia [106]. Despite this, clinical applications and therapeutic effects are compromised due to its low bioavailability, short half-life, and high hydrophilic nature, which requires high doses for effective treatment, resulting in several side effects [107,108]. In the work proposed by Li et al., a new system has been developed for the treatment of gastric cancer. In particular, the DOX was first loaded into the HNTs and then the DOX-HNTs were encapsulated in soy phospholipids to obtain DOX-HNTs-LIP. The LIP shell plays a protective role against DOX-HNT; furthermore, the nanocomposite showed high hemocompatibility, thanks to the protective function performed by the LIP. The release study, performed in vitro, showed that the system possesses a pH-sensitive release property, in fact, drugs were more easily released under acidic environments such as the tumor microenvironment. The in vivo and in vitro studies, reveal that HNTs/DOX/LIP complexes more significantly inhibit tumor growth than free DOX at the same drug concentration [109]. Another drug, considered to be among the mainly potent anticancer agent is the camptothecin (CPT), that shows great antitumor activity over a wide spectrum of human cancers [110]. Like other drugs, CPT has low water solubility and is toxic for non-tumour cells. For this reason, a folic acid–chitosan oligosaccharide/magnetic HNTs (FA-COS/MHNTs) has been developed as a CPT carrier, that was entrapped in the inner lumen of magnetic HNTs. The system showed a prolonged release of the drug for 60 h and a substantial inhibition of the growth of human colon carcinoma cells (Caco-2). Furthermore, thanks to the presence of folic acid (FA), this nanocomposite presents specificity in preferentially targeting tumour cells, thanks to an improved cellular uptake mediated by FA and COS [111]. The polymer–clay nanocomposite hydrogel films (PCNCHFs) were prepared from caboxymethyl cellulose, polyvinyl-pyrrolidone, agar and nano sepiolite clay, as reported in Palem et al. [112]. The PCNCHFs was conjugated with an anticancer drug 5-fluorouracil (FU) to obtain the nanocomposites PCNCHFs@FU. Analysis of the results showed that the nanocomposite possesses good biocompatibility and higher swelling capacity with sustained and stable FU release. Lisuzzo et al. proposed a new type of biohybrid material, a film prepared by co-assembling nanotubular halloysite, fibrous sepiolite, and cellulose nanofibers (HNT-SEP-CNF) in a 1:1:1 (*w*/*w*) ratio for the three components, to obtain homogeneous and self-supported films with excellent mechanical properties (Figure 4). This biohybrid material was evaluated as drug delivery systems using salicylic acid and ibuprofen. The results showed their encapsulation into the positively charged HNT lumen and more sustained release kinetics for the hybrid nanopaper as compared to the loaded neat halloysite. Furthermore, the antimicrobial activity against gram-positive and gram-negative bacteria was also evaluated, demonstrating an effective inhibition of *S. aureus* growth [113]. 

Among central nervous system disorders and brain disease, epilepsy is a common example of a neurological disorder. Drugs currently used for epilepsy treatment show a wide range of side effects which could be reduced by utilizing nanosized carriers that allow a targeted and slow-release drug delivery. Saleh et al., proposed the use of halloysite nanotubes to go through the blood–brain barrier and effectively deliver the payload over an extended time. They demonstrated that halloysite nanotubes (with fluorescent rhodamine B isothiocyanate) penetrate into primary rat brain microvascular endothelial cells (BMVECs), and were concentrated around the nuclei, showing their ability to act as drug carrier and delivery system [114]. 

### 4.2. Gene Delivery 

**Figure 5:**
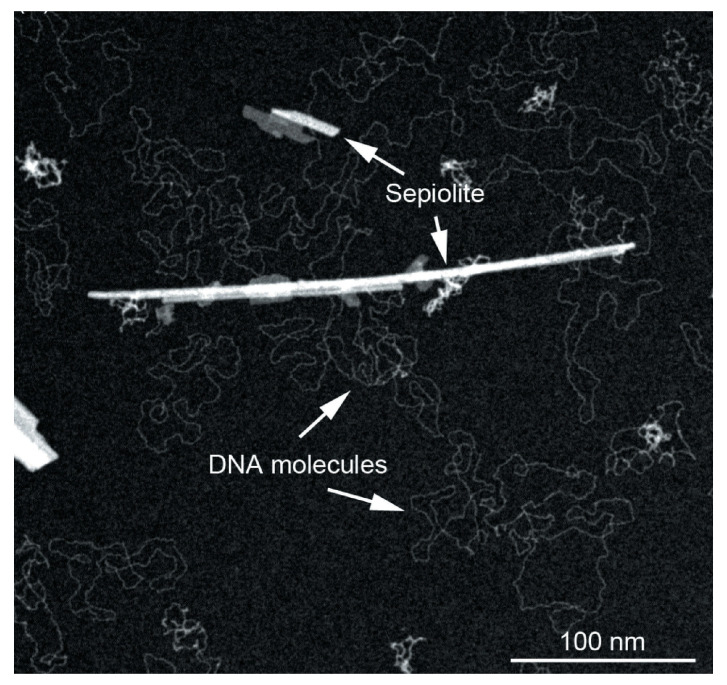
TEM image showing the strong interaction between sepiolite fibers and DNA molecules. Adapted from [74].

In addition to the use of chemotherapeutic drugs, gene therapy for cancer treatment has been widely investigated. It consists in the administration of exogenous genomic material in order to influence gene expression or to change the biological properties of cells for a therapeutic purpose [115]. For a long time, viral vectors have been used for the administration of nucleic acids, thanks to their capacity to transfer genes within human cells [116]. Viral vector-mediated insertional mutagenesis is an important risk associated with gene therapy. This can result in the disruption of the gene’s expression, with a consequent alteration in the expression of proto-oncogenes or tumour suppressor genes. For this reason, research has focused on the development of non-viral vectors that are characterized by better biosafety, limited immunogenicity and simplified preparation procedures [117]. Clay materials have shown promising potential as non-viral gene vectors [118,119]. The antisense oligodeoxynucleotides (ODNs) and small interfering RNA (siRNA) are widely used in anticancer gene therapy. The ODNs are short single-stranded DNA molecules constituted by few nucleotides that can bind to complementary regions of a target messenger RNA (mRNA), with the consequent inhibition of gene expression. siRNAs are small endogenous non-coding RNAs made up of about 21 nucleotides, and they have the ability to inhibit the expression of specific target genes [120]. Shi et al., presented the HNTs functionalization with APTES ((3-Aminopropyl) triethoxysilane) to facilitate the loading and administration. The obtained results showed that this system had good capacity for intracellular administration (98.7%) of the ODNs and an excellent ability to improve the antitumor potential of the ODNs towards HeLa cells [121]. Wu et al. have synthesized HNTs modified with the Polyethylenimine (PEI) for the delivery of anti-survival siRNA in pancreatic cancer cells (PANC-1), with the aim of reducing the levels of the survival protein that is responsible to inhibit apoptosis and stimulate the proliferation of cancer cells. Furthermore, mercaptoacetic acid-capped (CdSe) quantum dots were linked by a non-covalent bond to the anti-survival siRNA to then be linked to the PEI-HNT complexes with a resulting excellent considerable transfection efficiency in PANC-1 cells. The in vitro cytotoxicity studies showed an increase in apoptosis and in the antitumor potential of anti-survival siRNA. After a 72 h treatment with the complex, western blot analysis showed a 90% reduction in target protein levels in PANC-1 cells, confirming the system’s ability to silence the surviving gene [122]. HNTs grafted with a poly (amidoamine) dendrimer (PAMAM) were synthetised for the intracellular administration of siRNAs which target the gene-encoding vascular endothelial growth factor (VEGF). The HNT-PAMAM/siRNA complex showed a high efficiency of cellular uptake (94.3%) and an elevated biocompatibility. The VEGF expression decreased by 78% and induced apoptosis in MCF-7 cells (human breast cancer). Through in vivo studies was demonstrated that HNT-PAMAM/siRNA complex was able to reduce tumour volume by 55.1% and to inhibit angiogenesis, suggesting that it could represent a promising strategy in breast cancer gene therapy [119]. In addition to HNTs, the ability of the natural magnesium silicate clay mineral sepiolite to bind DNA makes it a potentially useful tool for biotechnological application. The interaction between DNA and sepiolite depends mainly on the sepiolite nanostructure and surface properties. Several studies have shown that DNA could bind efficiently to sepiolite. Thanks to its biocompatibility and ability to be spontaneously internalized and excreted by mammalian cells, sepiolite represents a good alternative for use as a non-viral vector for DNA. Furthermore, sepiolite possesses spontaneous fluorescence and this allows it to be detected in cells without the requirement of fluorescent chemical grafting [92]. It has been observed that natural raw sepiolite appears at the electronic microscope as bundles resulting from the agglomeration of their silicate microfibers. These can be disaggregated in concentrated water dispersions by applying high-speed mechanical shearing or by ultrasonication. The dispersion of aggregates can improve efficacy regarding different applications, for example, in DNA transfer into mammalian cells [31]. Because deagglomeration and dispersion of sepiolite nanofibers could also influence the toxicity of sepiolite in mammalian cells, Brooks et al. studied the process in order to identify the best conditions to produce detangled sepiolite for future biological applications. Sepiolite (from Vicálvaro-Vallecas, Madrid) was sonicated for different times and to investigate if sepiolite dispersion parameters impact the binding of biological macromolecules, was measured the adsorption isotherms for sonicated sepiolite (sSep) for protein (bovine serum albumin, BSA) or DNA. The results showed that while BSA protein adsorption increases with higher sonication time, the DNA adsorption does not show significant difference with increasing sonication time. Taking into account that ultrasonication improves the binding of biological macromolecules and reduces the cell toxicity, sepiolite could be very interesting for use in biomedical applications [15]. As mentioned above, sepiolite, thanks to its strong interaction with DNA molecules (Figure 5) and its ability to be naturally internalized into mammalian cells, can be used as a nanocarrier for DNA transfer [74].

The release of the DNA linked to sepiolite (from Vallecas-Vicálvaro, Madrid) has been evaluated with the biohybrids Sep + DNA placed in a buffer with a chelating agent (EDTA, ethylenediaminetetraacetic acid) supposing that that DNA desorption could occur by chelation of the cation bridges between the DNA molecules and the sepiolite surface. It was observed that DNA desorption was obtained with biohybrids Sep/DNA initially prepared in the presence of 5 mM MgCl_2_ and re-suspended in 10 mM Tris-HCl pH 7.5 and 5 or 10 mM EDTA. Furthermore, the quality of desorbed DNA was estimated, and the preservation of the DNA structure confirms that sepiolite could be a suitable support for the DNA, without altering its structure [31].

### 4.3. Wound Healing

Nanomaterials, thanks to their physicochemical properties, are perfect candidates to enhance wound healing as vehicles/carriers for controlled drug delivery, especially thanks to their higher surface/volume ratio [123]. Among nanomaterials, nanoclays have intrinsic properties such as good biocompatibility and degradation that possess remarkable potential for biomedical applications and for use in improving wound healing systems. It is estimated that chronic wounds affect around 6.5 million people annually and in patients with diabetes and other pathologies, chronic wounds have emerged as a major cause of mortality; hence, the need to develop novel and innovative approaches to accelerate wound healing [124]. The skin, being the principal external barrier, is continually exposed to external insult and acts as a primary defence barrier preventing internal structure from mechanical, thermal, and chemical damage [125]. The wound healing is a dynamic and complex physiological process involving various cell types, growth factors, proteinases, components of the extracellular matrix, mediators, and cytokines. This process is generally known as the rapid mechanism evolved by skin to close breaches to its barrier and consists of four phases: haemostasis, inflammation, proliferation, and dermal remodelling [126,127,128]. Actually, in the medical and economical field, skin wounds are of rising importance due to the growth of chronic diseases (for example, vascular disease, diabetes), an aging society and an elevated incidence of antibiotic resistance [129]. To manage wound healing, topical localized therapies are preferred because it avoids systemic effects. Moreover, with topical wound healing approaches, the wound site is covered and this help to prevent secondary infections [130]. Since conventional topical treatment for wound healing has different limitations, for example, allergic reaction, risk of infection or rapid drying, the nanotechnology could represent a promising approach to address the specificity and complexity of acute and chronic wounds [124,127,131]. 

Thanks to absorption abilities and stability under different pH conditions, the sepiolite constitutes a great tool for topical application. Dutta et al. reported the preparation of chitosan/sepiolite (CS-SEP) nanocomposite films for wound healing application. The antibacterial activity of chitosan (control) and CS-SEP (optimized) nanocomposite film was examined against gram-positive (*B. subtilis*) and gram-negative bacteria (*E. coli*). The result showed that the nanocomposite film exhibits better antibacterial activity against both the tested bacteria compared to control and possesses the properties required for wound healing application [132]. The increase of multidrug-resistant (MDR) pathogenic bacteria led the research toward the study of new methodologies to improve the physical, mechanical, and antibacterial properties of wound dressings treatments. Noor et al. proposed a nanocomposite hydrogel comprising cuprous oxide nanoparticles grafted sepiolite (Cu_2_O/Sep) and poly (vinyl alcohol) (PVA). The antibacterial properties were evaluated against *E. coli*, *S. aureus*, methicillin-resistant *S. aureus*, multidrug-resistant *P. aeruginosa*, and multidrug-resistant *K. pneumoniae* bacterial strains. The results showed that the addition of Cu_2_O/Sep within PVA hydrogel as well as increasing mechanical properties, enhanced its moisture retention capacities and swelling ratio. The antibacterial assay showed that the hydrogel effectively killed the multidrug-resistant bacteria [133]. The coagulation mechanisms of HNTs have been evaluated on HNTs-coated polyethylene terephthalate (PET) dressing fiber. The HNTs coating enhanced the resistance against liver and vessel bleeding and epidermal hemorrhage. The HNTs-PET dressing, prepared by the impregnation method, in addition to control hemorrhaging and skin bleeding in animal models, showed it was able to hinder the formation of a tight link between the clot and dressing fiber, thus avoiding wound adhesion [134]. Kouser et al. proposed for the first time HNT surface-modified with chitosan and incorporated in polycaprolactone (PCL) matrix for wound healing. The HNTs addition to the film increased roughness, mechanical strength, and enzymatic degradation. Furthermore, the nanocomposite showed enhanced cell proliferation, adhesion, and migration at mouse fibroblast cells as well as a good hemocompatibility with human erythrocytes [135].

### 4.4. Antimicrobial Activity

Antimicrobial agents present several formulations and are used in different applications, for example, in food packaging and medical equipment. The production of materials with antibacterial properties is growing year-by-year and industries need to ensure the safety of antimicrobials products [136]. 

**Figure 6:**
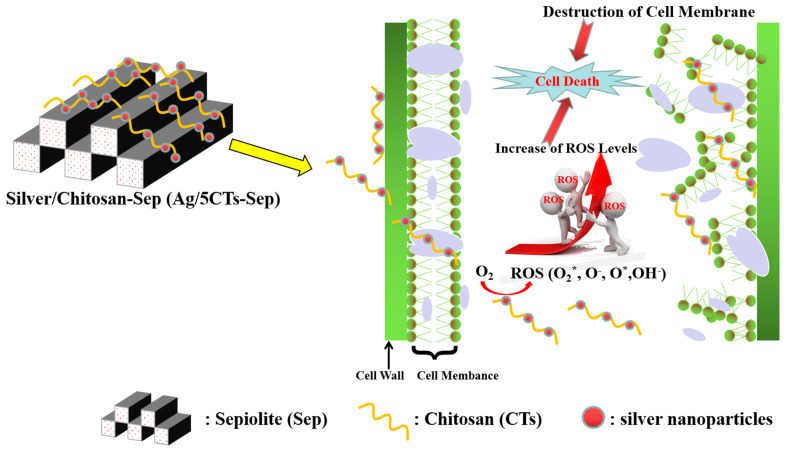
The possible synthetic and antifungal mechanisms of Ag/5CTs-Sep. Adapted from [137].

Natural clay minerals are recognized as the optimal inorganic carriers in antibacterial materials due to their excellent thermal stability, low cost and potent intercalation capacity. Li et al. present a hybrid material with low toxicity prepared with sepiolite (from Yuanyuan Sepiolite Technology Co., Ltd., Xiangtan city, Hunan, China), chitosan, and silver (Ag/5CTs-Sep) with antimicrobial activities. Its antimicrobial properties were investigated via the disk diffusion method and the results showed that the width of inhibition zone of Ag/5CTs-Sep against *S. aureus*, *E. coli* and *A. niger* reached 58.15, 32.95 and 35.18 mm, respectively. These great antimicrobial activities are due to the synergistic antimicrobial roles of CTs, Ag nanoparticles and the CTs-Ag composite structure. As regards the possible synthetic and antifungal mechanisms, they proposed the mechanism represented in Figure 6, in which the increase of reactive oxygen species (ROS) is due to the cell internalization of the antimicrobial components. ROS inside the cells can interact with DNA, RNA and protein disrupting normal cell metabolism and the cell membrane, leading to cell death [137]. 

**Figure 7:**
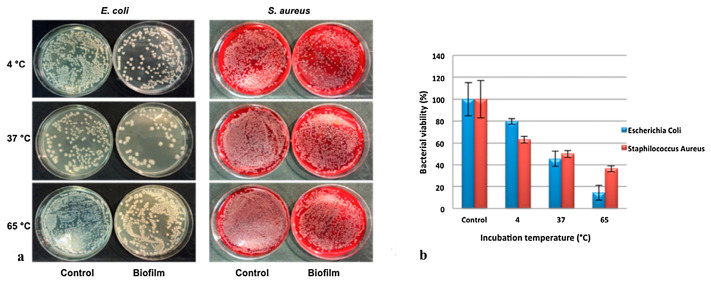
(**a**) Antibacterial activity of HNT/CB[6]/PO films against *E. coli* and *S. aureus* at 4, 37 and 65 °C. (**b**) *E. coli* and *S. aureus* viability expressed as percentage of bacterial viability.Adapted from [139].

The antibacterial activity of organic–inorganic composite based on a sepiolite–chitosan–zinc system was tested against *E. coli* and *S. aureus* and evaluated by the disk diffusion method. The composites were synthesized via different combination styles and named as chitosan–zinc oxide/sepiolite (CTs-ZnO/Sep), zinc oxide/chitosan–sepiolite (ZnO/CTs-Sep) and chitosan/zinc oxide–sepiolite (CTs/ZnO-Sep). The results showed that the sample presented different inhibition zone width against bacteria and that CTs-ZnO/Sep, ZnO/CTs-Sep and CTs/ZnO-Sep displayed better against *S. aureus* antibacterial activity than *E. coli* [138]. Biddeci et al. present a bio-nanocomposite film with both antioxidant and antimicrobial activities prepared by the filling of a pectin matrix with modified halloysite nanotubes containing the essential peppermint oil (PO). The HNTs surfaces were functionalized with cucurbit[6]uril (CB [6]) molecules, then, a pectin + HNT/CB [6] biofilm was prepared by the use of the casting method under specific experimental conditions in order to favor the entrapment of the volatile PO into the nanocomposite structure. The in vitro antibacterial activity of the biofilm was tested at specific temperatures (4, 37 and 65 °C), against gram-negative (*E. coli*) and gram-positive (*S. aureus*). Results showed that the cell viability for both bacterial strains on the biofilm loaded with PO showed a relevant antibacterial efficiency against *E. coli* and *S. aureus* strains (Figure 7a). The percentage of cell viability was reduced at 65 °C compared to those at 37 °C and 4 °C for both the strains (Figure 7b). The antimicrobial activity observed for *E. coli* at each incubation temperature was greater than *S. aureus* [139].

Among different active agents, ZnO nanoparticles possess promising antimicrobial properties; to this end, the synthesis and characterization of ZnO deposited halloysite nanotubes (ZnO@HNT) and incorporation of ZnO@HNT into PLA (polylactic acid) matrix as reinforcing nanofillers was presented by Boro et al. [140]. The antimicrobial efficacy of PLA-based nanocomposite films was determined against a gram-positive (*L. monocytogenes*) and a gram-negative (*E. coli*) bacteria by viable colony count method. The results showed that pristine PLA did not possess any antimicrobial activity towards *E. coli* and that the addition of HNT in PLA matrix did not show any antibacterial activity against both *E. coli* and *L. monocytogenes*. The inhibition on bacterial growth was affected by the presence of ZnO on the HNT surface (ZnO@HNT) and both incubation period and concentration of ZnO@HNT; in fact, the nanocomposite films with 2 wt% of ZnO@HNT showed reduction in bacterial vitality within 24 h of incubation period, and it further reduced to > 99% after 48 h. With higher loading (3 wt%) of ZnO@HNT, the antimicrobial effect was achieved within just 12 h of incubation period. Furthermore, the incorporation of ZnO@HNT in PLA matrix (PLA/ZnO@HNT) improves the physio-chemical properties and also imparts antimicrobial properties [140].

## 5. Conclusions

Clay minerals have attracted great interest since they are biocompatible materials with interesting properties. Among the various clay minerals, sepiolite and halloysite have been widely investigated for their use in several fields. In this review, we present the recent developments of the clay minerals research, focusing on their application as carrier for the delivery and the sustained release of biological active species, with particular attention to biomedical applications, such as drug delivery, tissue engineering, wound healing, and cancer therapy. The functionalization of clays surfaces, by means of supramolecular interactions or covalent modifications, opens different ways to obtain attractive nanomaterials which exhibit superior biological properties with respect to the unmodified ones. Research is ongoing to explore new applications of clay minerals in medicine. As is evident from what has been said, halloysite and sepiolite can be tailored to specific applications, can allow for precise delivery of therapeutic agents, and can enable the development of nano-scaffolds with enhanced properties that in the future can be used for regenerative medicine. In addition, they have an extremely high surface-area-to-volume ratio, allowing them to carry a large amount of drugs with minimal volume. In conclusion, we can say that the unique characteristics of clays make them perfect candidates to deliver drugs to targeted tissues, release drugs over a prolonged period of time, and protect drugs from environmental degradation. Although the biocompatibility of these materials has been demonstrated in several studies, it becomes clear that it is necessary to clarify the in vivo outcomes of long and chronic exposure, which seems to depend on different intrinsic characteristics of the materials. This makes us assume that over the next few years research efforts could be towards the development of cost-effective and highly biocompatible clay nanomaterials for their application in the biomedical field.

## Figures and Tables

**Table 1:** Main physical characteristics of sepiolite.

	Sepiolite	Reference
Chemical formula	Mg_8_(OH_2_)_4_[Si_6_O_15_]_2_(OH)_4_·8H_2_O	[20]
Length	2–10 µm	[19]
Width	10–30 nm	[21]
Thickness	5–10 nm	[21]
Specific surface area	80–350 m^2^/g	[21]

**Table 2:** Main physical characteristics of halloysite.

	Halloysite	Reference
Chemical formula	Al_2_Si_2_O_5_ (OH)_4_·nH_2_O	[45,46]
Length	2–10 µm	[54]
Internal diameter	10–30 nm	[53]
Internal diameter	40–70 nm	[53]
Specific surface area	65 m^2^/g	[55]

## Data Availability

Not applicable.
